# Association of CYP2A6 activity with lung cancer incidence in smokers: The multiethnic cohort study

**DOI:** 10.1371/journal.pone.0178435

**Published:** 2017-05-25

**Authors:** Sungshim L. Park, Sharon E. Murphy, Lynne R. Wilkens, Daniel O. Stram, Stephen S. Hecht, Loïc Le Marchand

**Affiliations:** 1 Department of Preventive Medicine, Norris Comprehensive Cancer Center, Keck School of Medicine, University of Southern California, Los Angeles, California, United States of America; 2 Masonic Cancer Center, University of Minnesota, Minneapolis, Minnesota, United States of America; 3 Epidemiology Program, University of Hawaii Cancer Center, Honolulu, Hawaii, United States of America; Legacy, Schroeder Institute for Tobacco Research and Policy Studies, UNITED STATES

## Abstract

While smoking is the primary cause of lung cancer, only 11–24% of smokers develop the malignancy over their lifetime. The primary addictive agent in tobacco smoke is nicotine and variation in nicotine metabolism may influence the smoking levels of an individual. Therefore, inter-individual variation in lung cancer risk among smokers may be due in part to differences in the activity of enzymes involved in nicotine metabolism. In most smokers, cytochrome P450 2A6 (CYP2A6)-catalyzed C-oxidation accounts for >75% of nicotine metabolism, and the activity of this enzyme has been shown to correlate with the amount of nicotine and carcinogens drawn from cigarettes. We prospectively evaluated the association of urinary biomarkers of nicotine uptake (total nicotine equivalents [TNE]) and CYP2A6 activity (ratio of urinary total trans-3′-hydroxycotinine to cotinine) with lung cancer risk among 2,309 Multiethnic Cohort Study participants who were current smokers at time of urine collection; 92 cases were diagnosed during a mean follow-up of 9.5 years. We found that higher CYP2A6 activity and TNE was associated with increased lung cancer risk after adjusting for age, sex, race/ethnicity, body mass index, smoking duration, and urinary creatinine (p’s = 0.002). The association for CYP2A6 activity remained even after adjusting for self-reported cigarettes per day (CPD) (Hazard Ratio [HR] per unit increase in log-CYP2A6 activity = 1.52; p = 0.005) and after adjusting for TNE (HR = 1.46; p = 0.01). In contrast, the association between TNE and lung cancer risk was of borderline statistical significance when adjusted for CPD (HR = 1.53; p = 0.06) and not statistically significant when further adjusted for CYP2A6 activity (HR = 1.30; p = 0.22). These findings suggest that CYP2A6 activity provides information on lung cancer risk that is not captured by smoking history or a (short-term) biomarker of dose. CYP2A6 activity should be further studied as a risk biomarker for smoking-related lung cancer.

## Introduction

Lung cancer is the second most common cancer in men and women, and the most common cause of cancer death [1]. Approximately 90% of lung cancer cases in the United States can be attributed to tobacco smoking [2]. However, only 11–24% of smokers will develop lung cancer over their lifetimes [2], and for the same self-reported number of cigarettes per day (CPD), lung cancer risk differs across racial/ethnic groups [3]. Nicotine is the primary addictive agent in tobacco smoke and these differences in risk have been suggested to be in part due to genetic variation in nicotine metabolism [4].

In most smokers, cytochrome P450 2A6 (CYP2A6)–catalyzed C-oxidation accounts for >75% of nicotine’s metabolism [5, 6]. The product of this pathway, cotinine, is then converted by CYP2A6, to *trans-*3′-hydroxycotinine (3-HCOT). Quantifying the sum of 3-HCOT and its glucuronide conjugate (referred to as "total 3-HCOT") captures the total conversion of cotinine to 3-HCOT. The ratio of total 3-HCOT to cotinine is a measure of CYP2A6 activity [5], which has been shown to influence smoking intensity [7, 8]. Smokers with reduced CYP2A6 activity tend to smoke less intensively, drawing in lower doses of nicotine and carcinogens from each cigarette, than do smokers with higher CYP2A6 activity because the former have more residual nicotine in their systems [7–10]. The dose of nicotine inhaled by a smoker may be quantified by measuring urinary total nicotine equivalents (TNE), which is the sum of total nicotine, total cotinine, total 3-HCOT and nicotine N-oxide. "Total” refers to the sum of the compound and its glucuronide conjugate. TNE is an excellent biomarker of short-term internal smoking dose since it captures 85–90% of nicotine uptake [5, 6]. A recent publication from the Shanghai Men’s Health study reported that *CYP2A6* genetic variants confirmed to be associated with a reduced enzymatic activity were associated with a decreased risk of lung cancer (odds ratio [OR] = 0.64; p = 0.03) [11]. This association was no longer statistically significant after accounting for TNE (OR = 0.74; p = 0.18), suggesting that the observed genetic association with lung cancer was the result of the relationship between nicotine metabolism and smoking dose.

The majority of studies that have been conducted have found that *CYP2A6* variants that result in reduced or no enzymatic activity are inversely associated with lung cancer risk [11–19]. However, many of these variants are specific to some populations (e.g. *4 is common in Japanese but rare in whites) and these genetic polymorphisms explain only a fraction of the variation in CYP2A6 activity (~44–64% depending on the ethnic/racial group) [9]; therefore, the phenotypic biomarker for CYP2A6 activity is expected to constitute a more suitable predictor of lung cancer risk. However, to date, there is no published data prospectively evaluating the association of a biomarker of nicotine metabolism with lung cancer risk. To determine whether such a biomarker captures information on lung cancer risk in current smokers beyond that provided by self-reported smoking history and internal smoking dose, we prospectively examined the association of a urinary biomarker of CYP2A6 activity with lung cancer risk among smokers in the Multiethnic Cohort (MEC) study.

## Materials and methods

### Study population

Subjects were participants in the MEC study, a prospective cohort study investigating the association of lifestyle and genetic factors with chronic diseases in five ethnic/racial populations (African Americans, Japanese Americans, Latinos, Native Hawaiians, and Whites) from Hawaii and Los Angeles, California [20]. This study comprised 215,251 men and women between the ages of 45 and 75 years at time of recruitment. In 1993–1996, each participant completed a mailed, self-administered questionnaire regarding demographic, dietary and other lifestyle exposures. The questions used in this study have undergone rigorous pilot testing and have been validated in a multiethnic test population at the time of design of the MEC study [20]. Briefly, pre-testing activities began with face-to-face administration of early drafts by trained interviewers. After revisions to improve awkward wording and layout, a mailed questionnaire was distributed to 130 subjects and later expanded to 1,000 randomly selected subjects of each racial/ethnic group. Reproducibility of the questionnaire was tested approximately 6 months later in about 300 subjects. Specifically, in relation to smoking, we found that the gold standard biomarker for internal smoking dose, total nicotine equivalents (which accounts for >85% of nicotine uptake), was significantly correlated with self-reported smoking dose, cigarettes per day (partial correlation, adjusted for age, sex, and race/ethnicity r = 0.50).

Approximately 10 years after baseline, a blood sample and an overnight (in Hawaii) or first morning (in Los Angeles) urine sample were collected from over 67,000 MEC participants. A short questionnaire was administered to update cigarette smoking exposure during the previous two weeks. All urine was kept on ice until processing. Aliquots were subsequently stored at -80°C until analysis. Approval for this study, including the consent procedure, was obtained from the Institutional Review Boards of the University of Minnesota, the University of Hawaii, and the University of Southern California. Study participants provided written consent.

IRB Code Number: 0912M75654. The present study includes 2,384 participants in the Biospecimen subcohort who were lung cancer-free and current smokers at the time of sample collection [6]. Baseline characteristics of participants, such as the average number of CPD and smoking duration were similar to those of the entire MEC population within each sex and racial/ethnic group [20].

### Epidemiologic data

The epidemiologic data used in this analysis, including information on smoking history, was obtained from a questionnaire administered at time of urine collection. Smoking duration and intensity at time of urine collection was collected using the following respective questions: “what is the total number of years you smoked” and “what is the average number of cigarettes smoked per day for the past two weeks” (prior to the date of urine collection). The data from the self-administered baseline and 10-year follow-up questionnaires were also used to impute body mass index (BMI) and CPD when missing at time of urine collection or to compute smoking duration [6]. Only one participant had no smoking duration information in the database and was excluded from the analysis.

### Biomarkers of smoking dose and nicotine metabolism

TNE was quantified as the sum of the urinary concentrations of total nicotine, total cotinine, and total 3-HCOT and nicotine N-oxide, where “total” refers to the sum of the metabolite and its glucuronide conjugate. CYP2A6 enzymatic activity was quantified as the ratio of total 3-HCOT/cotinine. All biomarkers were measured in all MEC smokers who provided a urine sample using liquid chromatography tandem mass spectrometry on urine samples [6]. The coefficient of variation for 22 undiluted samples from 10 blind replicates provided with the MEC samples was: 9.9% for TNE and 8.2% for total 3-HCOT/cotinine. Because there are large differences in creatinine levels by urine collection method (overnight in Hawaii vs. first morning in Los Angeles) and across the racial/ethnic groups, TNE was expressed in nmol/ml as opposed to per mg of creatinine and the creatinine values were adjusted for in the multivariable model.

### Follow-up and case ascertainment

To evaluate the risk of lung cancer in current smokers, this study encompassed the full range of TNE and CYP2A6 activity by including all MEC current smokers who were part of the Biospecimen subcohort. TNE was used to confirm current smoking status. Subjects with TNE >1.27 nmol/ml (4-times the limit of quantitation) were excluded, as this would indicate that these participants were not current smokers at the time of urine collection. For this study, we only included subjects who had complete data for the main covariates: age, sex, race/ethnicity, CPD and smoking duration. This resulted in n = 2,309 eligible participants. Participants’ follow-up began at the time of urine collection, for which urinary biomarkers of CYP2A6 activity and TNE were measured, and continued until the first of the following endpoints: 1) diagnosis of lung cancer; 2) death; or 3) end of follow-up, December 31, 2012 for participants in Los Angeles or December 31, 2014 for participants in Hawaii. Incident cancer cases, defined by International Classification of Diseases for oncology (ICD-O-3) and ICD-10, C34, were identified through linkages of the cohort to the Hawaii and California tumor registries which are part of the Surveillance, Epidemiology and End Results (SEER) program of NCI. Deaths are identified through annual linkage to state death certificate files in California and Hawaii and periodically to the National Death Index. By the end of follow-up (average 9.5 years), 92 incident invasive primary lung cancer cases were identified. Among the lung cancer cases, the majority were diagnosed with distant stage (53%); squamous cell carcinoma and adenocarcinoma were the most common histological cell-types (31% and 29%, respectively), followed by other/unspecified (21%), small cell lung cancer (15%) and large cell lung cancer (4%). Missing information on stage (n = 24) and histology (n = 8) and the unspecified cell-types (n = 18) were the results of these cases being identified by the rapid reporting system of the SEER registries which does not immediately capture all relevant information.

### Statistical methods

Risk of lung cancer was estimated by the hazard ratios (HR) and 95% confidence intervals (CIs) using Cox proportional hazards models where age was the time metric and follow-up began at urine collection. To reduce the influence of outliers on the results, we log-transformed (natural log) the variables with long right hand tails: BMI, CPD, creatinine, CYP2A6 activity and TNE. Distribution plots for CYP2A6 activity and TNE can be found in Supplemental Materials. Analyses were adjusted for age at urine collection (years, continuous), sex, race/ethnicity, urinary log-creatinine, log-BMI, and smoking duration (years, continuous) (Model 1). To investigate potential residual confounding by racial admixture within this multiethnic population, the analysis was also carried out on a subset of 2,215 smokers with GWAS data [21] using a model adjusted for principal components of genetic ancestry [22]; findings were similar. To assess whether the biomarkers of nicotine uptake provided information on lung cancer risk beyond that provided by self-reported measures of smoking, Model 1 was further adjusted for self-reported CPD (Model 2). Finally, to assess independent effects of these biomarkers, as CYP2A6 activity has been found to influence smoking behavior [10] and dose [7, 8, 10], we adjusted the model for both biomarkers of CYP2A6 activity and smoking dose (Model 3). The Cox proportional hazards assumption was met for all variables of interest. Due to the small sample size, associations by tumor histology or specific race were not evaluated. All statistical analyses were performed in SAS version 9.4 (SAS Institute, Cary, NC).

## Results

Table 1 presents the baseline characteristics of this cohort of MEC smokers (n = 2,309) and the characteristics for the 92 incident lung cancer cases. Among the eligible population there was a greater proportion of Japanese Americans (31%) and women (54%) than among cases. The median age at urine collection was 63 years. As expected, those who developed lung cancer during follow-up had characteristics that correlated with an increase in lung cancer risk [23]. These participants were slightly older at the time of urine collection and reported smoking more CPD and for a longer duration. Also, those who became cases had higher levels of TNE (33.5 vs. 32.4 nmol/ml) and CYP2A6 activity (3.8 vs. 3.3). As expected from our previous study [7], higher CYP2A6 activity was associated with higher TNE even after adjusting for the covariates in Model 2 (age at urine collection, sex, race/ethnicity, log-BMI, smoking duration (years), log-urinary creatinine, and log-CPD) (partial correlation coefficient: 0.24, p<0.0001). S1 Fig presents density distribution plots for TNE and the CYP2A6 activity ratio both without and with log-transformation for the overall cohort and by lung cancer case-control status. Both biomarkers are slightly skewed to the right. Those who developed lung cancer during study follow-up presented with a slightly higher TNE and CYP2A6 activity levels than subjects who did not become a case during the same period of time.

**Table 1:** Characteristics of MEC current smokers at time of urine collection and incident lung cancer cases identified in this group during follow-up.

	Eligible population		Incident Lung cancer cases	
	(n = 2,309)		(n = 92)	
	**N**	**%**	**N**	**%**
**Ethnicity/Race**				
**African American**	367	16%	18	20%
**Native Hawaiian**	331	14%	21	23%
**White**	445	19%	20	28%
**Latino**	457	20%	7	8%
**Japanese American**	709	31%	26	22%
**Sex**				
**Male**	1067	46%	44	48%
**Female**	1242	54%	48	52%
	**Median**	**(interquartile range)**	**Median**	**(interquartile range)**
**Age at time of urine collection (yrs)**	63	(58–69)	66	(61–73)
**Cigarettes per day (CPD) at time of urine collection**	10	(6–20)	16	(10–20)
**Body mass index (BMI) at time of urine collection (kg/m**^**2**^**)**	25.6	(22.7–28.8)	24.4	(21.9–26.7)
**Follow-up time (yrs)**	9.5	(8.7–10.7)	6.6	(4.8–8.2)
**Pack-years**	23	(12–38)	35	(22–52)
**Age at smoking initiation (yrs)**	23	(19–29)	24	(21–26)
**Smoking duration (yrs) at time of urine collection**	41.5	(34.5–46.5)	46.5	(41.5–50.0)
**Total nicotine equivalents (TNE)**	32.4	(19.6–53.6)	33.5	(23.9–58.3)
**CYP2A6 activity**^a^	3.3	(1.80–5.59)	3.8	(2.05–6.09)

^a^ CYP2A6 activity is the urinary total 3-HCOT/cotinine ratio

Table 2 presents the associations of known risk factors with lung cancer. Consistent with previous findings in the entire MEC study [3], after accounting for age, sex, BMI, CPD and smoking duration, when compared to whites, African Americans and Native Hawaiians had a 2-fold (95% CI: 1.02–3.91) and 1.79-fold (95% CI: 0.96–3.34) increased lung cancer risk, respectively. CPD and smoking duration were both significantly associated with an increased lung cancer risk (per unit increase in log-CPD, HR = 1.71; 95% CI: 1.22–2.39 and per year increase in smoking duration, HR = 1.07; 95 CI: 1.03–1.11).

**Table 2:** Associations of various characteristics at time of urine collection with lung cancer incidence (n = 92 cases).

	HR^a^	(95% CI)^a^	p-value
**Female vs. Male**	1.01	(0.65–1.55)	0.979
**African American vs. White**	2.00	(1.02–3.91)	0.043
**Native Hawaiian vs. White**	1.79	(0.96–3.34)	0.067
**Latino vs. White**	0.50	(0.20–1.21)	0.123
**Japanese American vs. White**	0.88	(0.49–1.59)	0.672
**Body mass index (BMI) (natural log, kg/m**^**2**^**)**	0.30	(0.10–0.95)	0.040
**Cigarettes per day (CPD) (natural log)**	1.71	(1.22–2.39)	0.002
**Smoking duration (years)**	1.07	(1.03–1.11)	<0.0001

^a^ Hazard ratio from Cox regression, adjusted for age, sex, race/ethnicity, log-BMI, log-CPD and smoking duration

We found that after adjusting for age, sex, race/ethnicity, BMI, urinary creatinine levels, and smoking duration (Model 1), both TNE and CYP2A6 activity were associated with an increase in lung cancer risk (HR per log-TNE increase = 1.83; 95% CI: 1.24–2.71 and HR per log-CYP2A6 activity increase = 1.60; 95% CI: 1.20–2.14). With further adjustment for self-reported smoking dose (Model 2), CPD, CYP2A6 activity remained associated with lung cancer risk (HR per unit increase in log-CYP2A6 activity = 1.52; 95% CI: 1.14–2.04), whereas, the lung cancer association with TNE was only of borderline statistical significance (HR per log-TNE increase = 1.53; 95% CI: 0.99–2.37) (Table 3, Model 2). When adjusting for CYP2A6 activity, a biomarker found to influence smoking dose [7, 8, 10], the association with TNE was no longer statistically significant (lung cancer HR per unit increase in log-TNE = 1.30; 95% CI: 0.85–1.99). Alternatively, when further adjusting for TNE, log-CYP2A6 activity remained significantly associated with lung cancer (HR per unit increase in log-CYP2A6 activity = 1.46; 95% CI: 1.08–1.98). Moreover, when Japanese Americans, a population with a high frequency of the CYP2A6 null allele, *4, were removed from our analysis, the association for higher CYP2A6 activity ratio with lung cancer risk was still statistically significant in the remaining four racial/ethnic groups (HR = 1.53; 95% CI: 1.03–2.26).

**Table 3:** Associations of biomarkers of nicotine uptake (TNE) and nicotine metabolism (CYP2A6 activity) with lung cancer incidence (92 cases).

	Model 1^a^			Model 2^b^			Model 3^c^		
	HR	(95% CI)	p	HR	(95% CI)	p	HR	(95% CI)	p
Log-TNE (nmol/ml)	1.83	(1.24–2.71)	0.002	1.53	(0.99–2.37)	0.06	1.30	(0.85–1.99)	0.22
Log-CYP2A6 activity^d^	1.60	(1.20–2.14)	0.002	1.52	(1.14–2.04)	0.005	1.46	(1.08–1.98)	0.015

^a^ Model 1 includes age at urine collection, sex, race/ethnicity, log-BMI, smoking duration (years), and log-urinary creatinine.

^b^ Model 2 further includes log-CPD

^c^ Model 3 further includes log TNE or log-CYP2A6 activity

^d^ CYP2A6 activity is the urinary total 3-HCOT/cotinine ratio

## Discussion

This is the first study showing that a biochemical marker of nicotine metabolism is associated with lung cancer risk. We found that even after accounting for both self-reported smoking history (CPD and duration) and internal smoking dose (TNE), and other potential confounders, a one natural log-unit increase in the CYP2A6 enzymatic activity ratio (urinary total 3-HCOT/cotinine) was associated with a statistically significant 46% increased lung cancer risk. This result suggests that CYP2A6 activity may capture information about risk beyond that provided by smoking history.

In most smokers, at least 75% of the metabolism of nicotine occurs via CYP2A6-mediated C-oxidation [5, 6]. Prior studies have shown that CYP2A6 activity, measured by various metabolite ratios (e.g. total 3-HCOT/total cotinine, 3-HCOT/cotinine, and total 3-HCOT/cotinine) modifies internal smoking dose [7, 8, 10]. Individuals with low nicotine metabolism were found to smoke less intensively in order to achieve the same internal dose of nicotine, the addictive component in tobacco, compared to individuals who metabolize nicotine more rapidly [7, 8, 10]. In an earlier analysis examining the inter-relationships of the biomarkers under study in our MEC smokers population, we showed that CYP2A6 activity and genetic variants of CYP2A6 activity were positively associated with TNE and that this association was consistently observed in all five racial/ethnic groups [9]. In the present lung cancer analysis, when CYP2A6 activity was adjusted for, TNE was no longer associated with risk, suggesting that the enzymatic activity ratio more accurately captures a smoker’s long-term tobacco carcinogens exposure than does TNE, which only reflects short-term exposure. Additionally, lower CYP2A6 activity may result in a reduction in the activation of the lung carcinogen NNK by CYP2A6. CYP2A6 activity has been has been correlated with lower bioactivation, which would lead to lower levels of NNK-related DNA adducts and a potential decrease in tumor induction [24].

Previous studies have investigated the association of functional variants in the *CYP2A6* gene with lung cancer risk. Some studies reported that, consistent with our findings, low *CYP2A6* activity variants were associated with a reduced risk of lung cancer [14–16], possibly only in smokers [18]. However, other studies have shown that low activity variants were either not associated or positively associated with risk [12, 13, 17]. These inconsistencies may be the result of small sample sizes and difficulty in genotyping this region due to the high sequence homology (>85%) that exists between *CYP2A6* and *CYP2A7*, as well as the marked racial/ethnic differences in frequency for the functional variants[25].

In an earlier report, using a genome-wide association study approach in two independent studies, we showed that the top six *CYP2A6* variants associated with lung cancer risk (n = 13,479 cases and 43,218 controls) were also associated with CYP2A6 activity [19]. Consistent with our findings, a recent publication by Yuan et al. [11] found that candidate genotyping of four known poor metabolizing variants were associated with lower TNE levels and a 26% decreased risk of lung cancer. However, this study was comprised of Chinese, a population with a relatively high frequency of the *CYP2A6* deletion (*4) and the association of lung cancer risk with the *CYP2A6* genotype was no longer statistically significant when adjusted for TNE. Whereas, in the present prospective study of five racial/ethnic groups (with a range of frequencies for *CYP2A6* variant alleles), we observed a significant association for CYP2A6 activity with lung cancer risk, even after adjusting for TNE. Indeed, when Japanese Americans, a group with a >35% frequency of carrying at least one copy of the *CYP2A6* gene deletion (*4)[9], were removed from our analysis, the association for higher CYP2A6 activity with lung cancer was statistically significant in the remaining four racial/ethnic groups. These results suggest that the CYP2A6 activity biomarker (urinary total 3-HCOT/cotinine) may capture the net effect of various *CYP2A6* variants, which individually may each have only a modest effect on nicotine metabolism, and that of possible inducers that are typically not measured in genetic association studies.

The present study’s limitations include the modest number of lung cancer cases and inability to investigate risk by tumor cell-type or race/ethnicity. Replication in a prospective study with a larger number of lung cancer cases is warranted. The study strengths include the prospective design, the racial/ethnic diversity of our sample, our ability to adjust for confounders, and our comprehensive, biomarker-based measurements of the metabolic phenotype and exposure of interest.

In conclusion, this study suggests that a urinary marker of nicotine metabolism is associated with incidence of lung cancer in smokers, even after taking into account self-reported smoking history or a short-term biomarker of dose. Thus, measuring CYP2A6 activity may improve lung cancer risk prediction in smokers. Additional prospective analyses with a larger sample size are warranted to confirm these findings.

## Supporting information

S1 Fig Density distribution plots for TNE and the CYP2A6 activity ratio both without and with log-transformation for the MEC smokers and by the participants’ lung cancer case-control status. Figure A presents the density distribution plot for TNE without log-transformation; figure B presents the density distribution plot for TNE with log-transformation; figure C presents the density distribution plot for CYP2A6 without log-transformation; and figure D presents the density distribution plot for CYP2A6 with log-transformation.
(PDF)
