# Supplementary material for: Association of CYP2A6 activity with lung cancer incidence in smokers: The multiethnic cohort study
Source: PLoS One. 2017 May 25;12(5):e0178435. doi: 10.1371/journal.pone.0178435 (PMC5444837; doi:10.1371/journal.pone.0178435)

Supporting information

S1. Fig. Density distribution plots for TNE and the CYP2A6 activity ratio both without and with log-transformation for the MEC smokers and by the participants' lung cancer case-control status.

A.

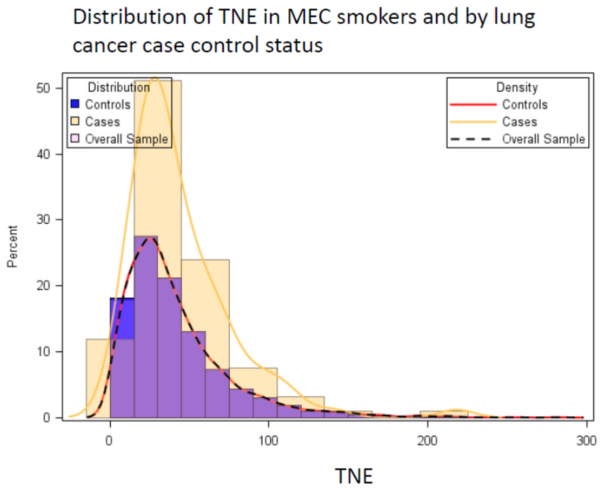

B.

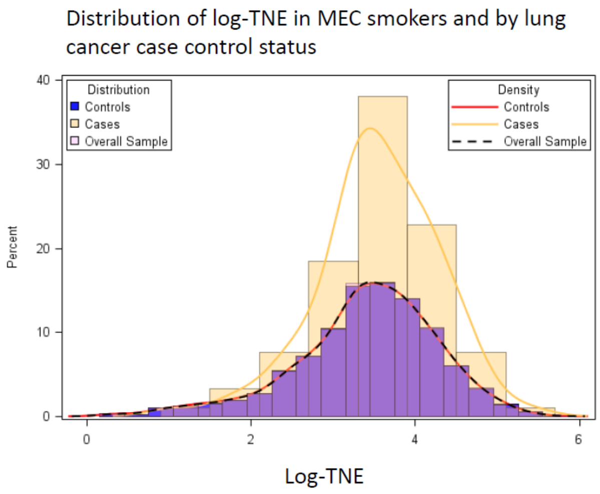

C.

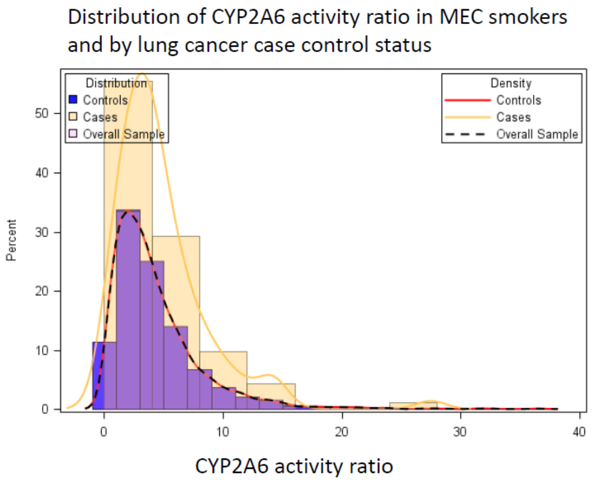

D.

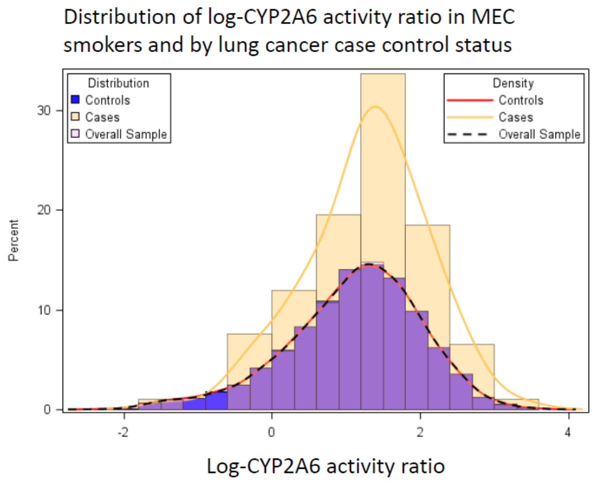

Supplement: S1 Fig — Figure A presents the density distribution plot for TNE without log-transformation; figure B presents the density distribution plot for TNE with log-transformation; figure C presents the density distribution plot for CYP2A6 without log-transformation; and figure D presents the density distribution plot for CYP2A6 with log-transformation. (PDF) [file pone.0178435.s001.pdf]
